# Citrate pretreatment promotes rice (*Oryza sativa* L.) coleoptile elongation under submergence

**DOI:** 10.5511/plantbiotechnology.24.1220a

**Published:** 2025-03-25

**Authors:** Akio Kubo, Miho Sanagi, Yuko Maki, Ryosuke Koyari, Futoshi Sakuma, Junji Yamaguchi, Takeo Sato

**Affiliations:** 1 Graduate School of Life Science, Hokkaido University, Sapporo, Hokkaido 060-0810, Japan; 2 Faculty of Science, Hokkaido University, Sapporo, Hokkaido 060-0810, Japan; 3 Creative Research Institution, Hokkaido University, Sapporo, Hokkaido 060-0810, Japan; 4 Snow Brand Seed Co. Ltd., Naganuma, Hokkaido 069-1464, Japan

**Keywords:** anaerobic metabolism, coleoptile elongation, organic acid, rice, submergence

## Abstract

Oxygen depletion due to submergence causes cellular energy starvation and severely restricts the growth of most plant species. To survive hypoxic and anoxic environments under submergence, rice (*Oryza sativa* L.) possesses various adaptive mechanisms including energy production from seed storage starch via anaerobic respiration and coleoptile elongation during early post-germinative growth. However, further investigation of the submergence tolerance mechanism is important for understanding its effect on plant physiology and agricultural production. Here, we found that pretreatment of rice seeds with organic acids, such as citrate and lactate, improved subsequent seedling growth under submergence. Citrate pretreatment promoted coleoptile elongation under submergence. Moreover, the expression of genes related to anaerobic respiration and phenylpropanoid biosynthesis was activated in the embryo of citrate treated seeds during submergence while the expression of genes encoding starch degradation enzymes and signaling factors was not significantly influenced. Accordingly, starch and soluble sugar amounts in the endosperm were not altered by citrate pretreatment. These results suggest that citrate pretreatment promotes coleoptile elongation in rice seeds under submergence via the transcriptional regulation of genes related to anaerobic energy production, possibly through an unknown mechanism related to phenylpropanoid metabolism.

## Introduction

Rice is an important food crop that feeds more than half of the global population (Lee et al. 2014). Given that rice production is a labor- and energy-consuming process, reducing the production cost is extremely important for ensuring a steady supply. Direct seeding, which involves the sowing of rice seeds directly in submerged soil, is an effective method for reducing production costs by cutting down on the manpower and time involved in production (Kumar and Ladha 2011). However, hypoxic conditions due to the submergence result in poor seedling establishment and consequently weak plants and low yield (Ma et al. 2020). Although hypoxia tolerant varieties and chemical treatments have been developed to improve seedling establishment under submergence (Zhang et al. 2023), these methods have been only partially successful.

Under submergence, seeds are subject to energy starvation due to hypoxia. However, rice seeds are able to germinate and produce a coleoptile under submergence (Perata and Alpi 1993), primarily using the energy produced by starch degradation in the endosperm and anaerobic metabolism in the embryo (Gibbs et al. 2000; Perata et al. 1993). The starch degradation enzyme α-amylase (1,4-α-D-glucan maltohydrolase) mediates the mobilization of stored starch, and RAmy3D is a major functional isoform of α-amylase in submerged germination (Damaris et al. 2019). The plant energy sensor SNF1-Related Kinase 1 (SnRK1) plays an important role in promoting *RAmy3D* gene expression through the activation of the MYBS1 transcription factor (Crepin and Rolland 2019; Lee et al. 2009; Lu et al. 2007). Alcohol dehydrogenases (ADH1 and ADH2) and pyruvate decarboxylases (PDC1 and PDC2) are involved in the alcoholic fermentation process, which leads to the reduction of pyruvate into alcohol (Gibbs et al. 2000; Takahashi et al. 2014; Vijayan et al. 2018). Energy starvation due to hypoxia activates the calcium signal (Lee et al. 2009). Calcineurin B-like (CBL) protein-interacting protein kinase 15 (CIPK15) plays a critical role in sensing low oxygen stress and sugar starvation (Lee et al. 2009). CIPK15 interacts with the Ca^2+^-binding CBL4 proteins, which act as a calcium ion sensor, and activates the SnRK1A-MYBS1-RAmy3D signaling module (Lee et al. 2009). In addition, CIPK15 activates alcoholic fermentation by upregulating *ADH* transcription under submergence (Lee et al. 2009). In addition to metabolic adaptation, the roots of rice plants exhibit changes in cell wall composition, with increased suberization and/or lignification, under submergence-induced hypoxia to avoid radial oxygen loss (ROL) (Colmer et al. 2019). Interestingly, a recent study suggested that metabolites produced by anaerobic microorganisms in the rhizosphere affect hypoxia stress responses in rice (Colmer et al. 2019). Organic acids stimulate ROL barrier formation (Colmer et al. 2019). However, the detailed molecular mechanism mediating the submergence response in rice remains unclear.

In this study, we investigated the effect of organic acid pretreatment of rice seeds on seedling establishment under submergence. We found that citrate pretreatment enhanced coleoptile elongation under submergence. Transcriptome analysis suggested that citrate pretreatment influences the expression of several key genes encoding anaerobic respiration enzymes and significantly upregulates the expression of phenylpropanoid biosynthesis genes.

## Materials and methods

### Plant materials and growth conditions

Seeds of *japonica* rice (*Oryza sativa* L. ssp. *Japonica*, cv. Nipponbare and Daichinohoshi) were sorted in 20% sodium chloride solution, sterilized with 5% sodium hypochlorite solution for 15 min, and washed 10 times with distilled water. Then, to carry out seed pretreatments, 6 g of seeds were incubated in 25 ml of distilled water (mock) or organic acid solution at 15°C in the dark for 4 days. Then, 15 seeds were transferred into a bottle, submerged in 100 ml of distilled water (at a depth of 9.5 cm), and incubated at 15°C in the dark for 2 weeks (for coleoptile elongation analysis and sugar and starch quantification) or 4 days (for RNA-seq and RT-qPCR analyses).

### Preparation of extract from lactic acid bacteria culturing medium (ELM)

*Lactiplantibacillus plantarum* strain HOKKAIDO was cultured in a modified MRS medium (with beef extract removed). The corn steep liquor (CSL) solution was purified using a strong basic anion exchange resin Diaion PA418 (Mitsubishi Chemical Co., Japan) and eluted with a solution containing 30% isopropanol and 3.7% hydrochloric acid. ELM was prepared by mixing the supernatant of *Lactiplantibacillus plantarum* strain HOKKAIDO culture medium and purified CSL solution in equal quantities (pH was adjusted to 4.0).

### Coleoptile elongation analysis

Coleoptile length was measured as the distance from the scutellum to the tip of the coleoptile. A total of 30 seedlings in each treatment (mock and organic acid) were analyzed.

### Reverse transcription-quantitative PCR (RT-qPCR) analysis

Total RNA was isolated from embryos using the TRIzol Reagent (Invitrogen, Waltham, MA, USA) and treated with RQ1 RNase-free DNase (Promega, Madison, WI, USA), according to the manufacturer’s instructions. First-strand cDNA was synthesized using the oligo(dT) primer (Promega) and SuperScript IV reverse transcriptase (Invitrogen). Then, qPCR was performed on the AriaMX system (Agilent Technologies, Santa Clara, CA, USA) using the TB Green premix EX Taq II (TaKaRa Bio Inc., Shiga, Japan) and sequence-specific primers (Supplementary Table S1).

### RNA-sequencing (RNA-seq) and data analysis

Total RNA was extracted from embryos using the RNeasy Plant Mini Kit (Qiagen, Hilden, Germany), and 600 ng of the extracted total RNA was used for library preparation. PolyA-selected and strand-specific libraries were prepared using the KAPA mRNA HyperPrep Kit (cat. no. KK8580, Kapa Biosystems, Wilmington, MA, USA) with the KAPA Universal adaptor (cat. no. 9063781001, Kapa Biosystems) and KAPA Unique Dual Index Primer Mixes (cat. no. 9134336001, Kapa Biosystems), according to the manufacturer’s protocol. All libraries prepared from three biological replicates per treatment were pooled and sequenced on the NovaSeq X sequencer (Illumina, San Diego, CA, USA) to obtain 150-bp paired-end reads. The quality control and pre-processing of raw paired-end reads were performed using fastp v0.19.5, an ultra-fast FASTQ preprocessor (Chen et al. 2018). Reads were aligned to the Nipponbare reference genome assembly (IRGSP-1.0) using STAR v2.7.10b (Dobin et al. 2013). The number of reads mapped to each gene was counted using featureCounts v2.0.1. (Liao et al. 2014). Differential expression analysis was performed using DESeq2 in R (Love et al. 2014), and genes with adjusted *p*-value<0.05 were identified as differentially expressed genes (DEGs). The Gene Ontology (GO) enrichment analysis of DEGs was performed using clusterProfiler v4.6.0 (Wu et al. 2021) in R. The transcripts per million (TPM) value of each gene was calculated using TPMCalculator v.0.0.3 (Alvarez et al. 2019).

### Quantification of starch and free sugar contents

The concentrations of starch and free sugars were determined as previously described (Aoyama et al. 2014). Briefly, ground samples were extracted twice by boiling in 0.5 ml of 80% ethanol for 5 min and separated into supernatant and pellet by centrifugation at 12,000×g for 15 min at 15°C. Then, free sugars in the embryos were quantified by treating the supernatant with the following enzymes in the presence of ATP and NAD^+^: 1 U of glucose-6-phosphate dehydrogenase (Sigma, St. Louis, MO, USA) and 1 U of hexokinase (Sigma) for glucose quantification; 2 U of phosphoglucose isomerase (Sigma) for fructose quantification; and 85 U of invertase (Sigma) for sucrose quantification. To quantify the starch content of endosperms, the pellet was dissolved in 0.5 ml of dimethyl sulfoxide, boiled for 15 min, and then digested with 1 U of α-amylase (Sigma) and 10 U of amyloglucosidase (Sigma). The absorbance of all samples was measured at 340 nm, the NADH specific peak.

## Results and discussion

### ELM pretreatment promotes rice seedling growth under submergence

Anaerobic germination tolerance is characterized by rapid coleoptile elongation under submergence. To understand the mechanism promoting submergence tolerance in rice, we explored the compounds that positively regulate coleoptile elongation. First, we examined whether the extract from lactic acid bacteria culturing medium (ELM), which has been reported to include plant growth-stimulating compounds (Jaffar et al. 2023; Maki et al. 2021, 2022), could promote rice coleoptile elongation under submergence. Rice seeds were incubated in water or diluted ELM for 4 days, and transferred to bottles and grown under completely submerged conditions in the dark. Nipponbare (*japonica* rice cultivar) seedlings generated from ELM-treated seeds showed longer coleoptiles than the control seedlings (Supplementary Figure S1). ELM pretreatment also promoted coleoptile elongation in Daichinohoshi (Supplementary Figure S2), an elite *japonica* rice cultivar developed for direct seeding in Japan (Kinoshita et al. 2006).

### Organic acid pretreatment promotes post-germination growth of submerged rice seedlings

**Figure 1:**
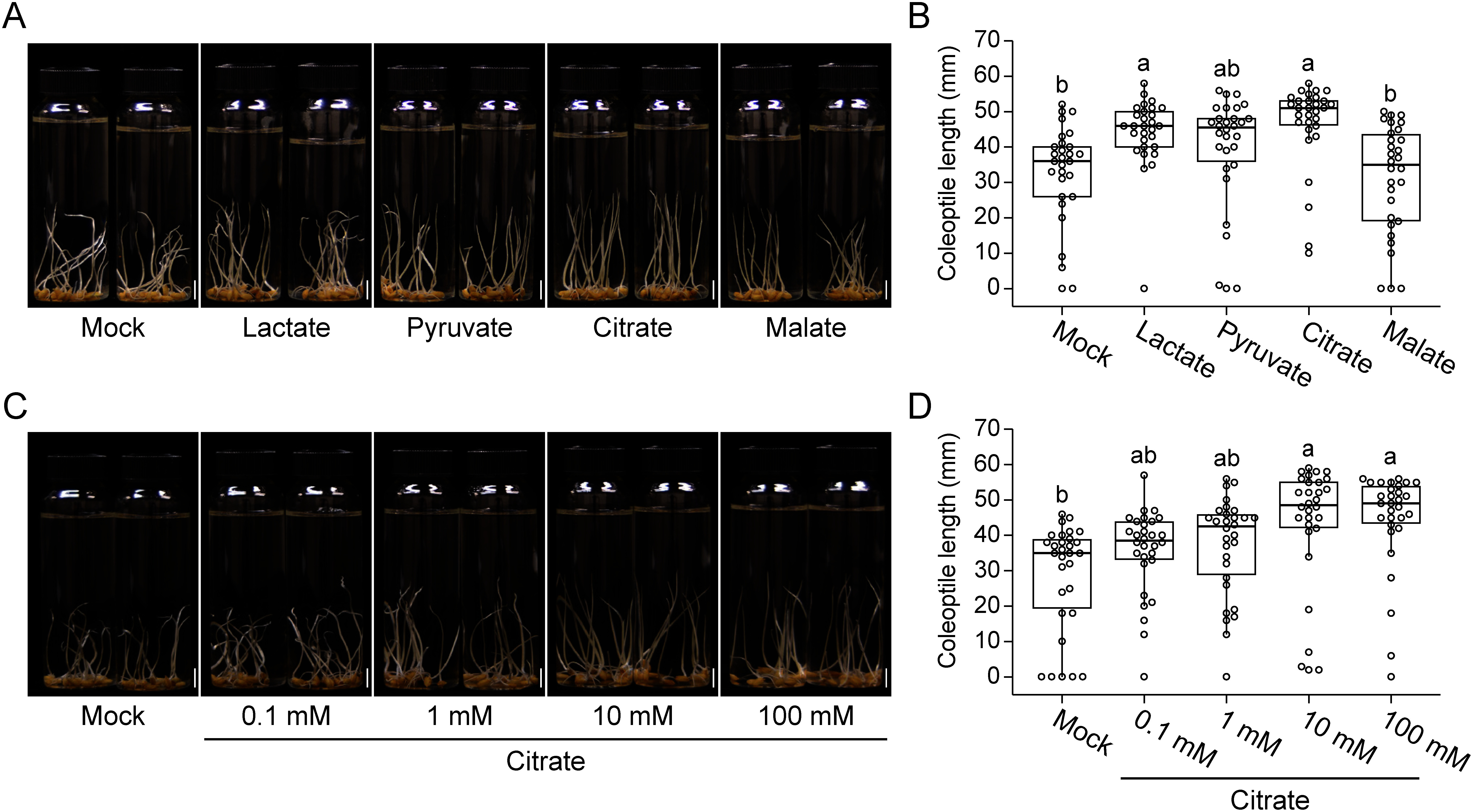
Figure 1. Organic acid pretreatment promotes rice coleoptile elongation under submergence. (A, B) Images (A) and coleoptile length (B) of 18-day-old Daichinohoshi seedlings pretreated with different organic acid solutions. Seeds were pretreated with distilled water (Mock) or 10 mM organic acid (Lactate, Pyruvate, Citrate, or Malate) for 4 days and then incubated in distilled water for 2 weeks. (C, D) Images (C) and coleoptile length (D) of 18-day-old Daichinohoshi seedlings pretreated with different concentrations of citrate. Seeds were pretreated with distilled water (Mock) or the indicated concentration of citrate for 4 days and then incubated in distilled water for 2 weeks. Scale bars=1 cm. In each box, the top and bottom lines represent the upper and lower quartiles, respectively; middle horizontal line represents the median (*n*=30); and whiskers indicate 1.5 times the interquartile range. Different letters indicate statistically significant differences (one-way ANOVA with Tukey’s HSD test, *p*<0.05).

We explored the chemical compounds related to the coleoptile elongation effect of ELM. A recent study showed that organic acids enhance the resistance to submergence stress in rice through the formation of the ROL barrier (Colmer et al. 2019). Since ELM contains a high amount of lactate, an organic acid related to anaerobic metabolism in microbes and mammals, we tested the effect of lactate pretreatment on coleoptile elongation in rice. Lactate pretreatment slightly promoted coleoptile elongation under submergence (Figure 1A, B). Then, we analyzed the effect of other organic acids related to anaerobic and aerobic carbohydrate metabolism, including pyruvate, citrate, and malate. Interestingly, citrate pretreatment significantly enhanced coleoptile elongation (Figure 1A, B). Like lactate, pyruvate also promoted coleoptile elongation in rice; however, malate did not show a promotional effect (Figure 1A, B). Next, we evaluated coleoptile elongation in seeds treated with various concentrations of citrate. The results showed that 10 mM citrate was sufficient for promoting coleoptile elongation (Figure 1C, D).

### Citrate pretreatment enhances the expression of anaerobic carbohydrate metabolism genes in rice embryos under submergence

**Figure 2:**
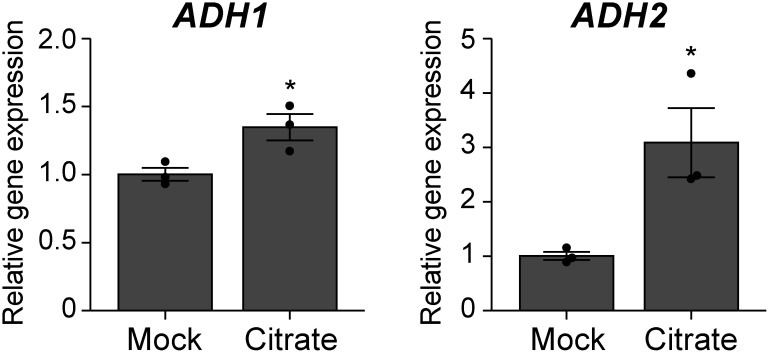
Figure 2. Citrate pretreatment enhances the expression of key anaerobic respiration-related genes in the embryos of submerged rice seeds. Daichinohoshi seeds were pretreated with distilled water (Mock) or 10 mM citrate and then incubated in distilled water for 4 days. Gene expression levels were first normalized to that of *UBC5b* and then represented relative to the mock sample. Data represent mean±SD (*n*=3). Asterisks indicate statistically significant differences (* *p*<0.05; Student’s *t*-test).

**Figure 3:**
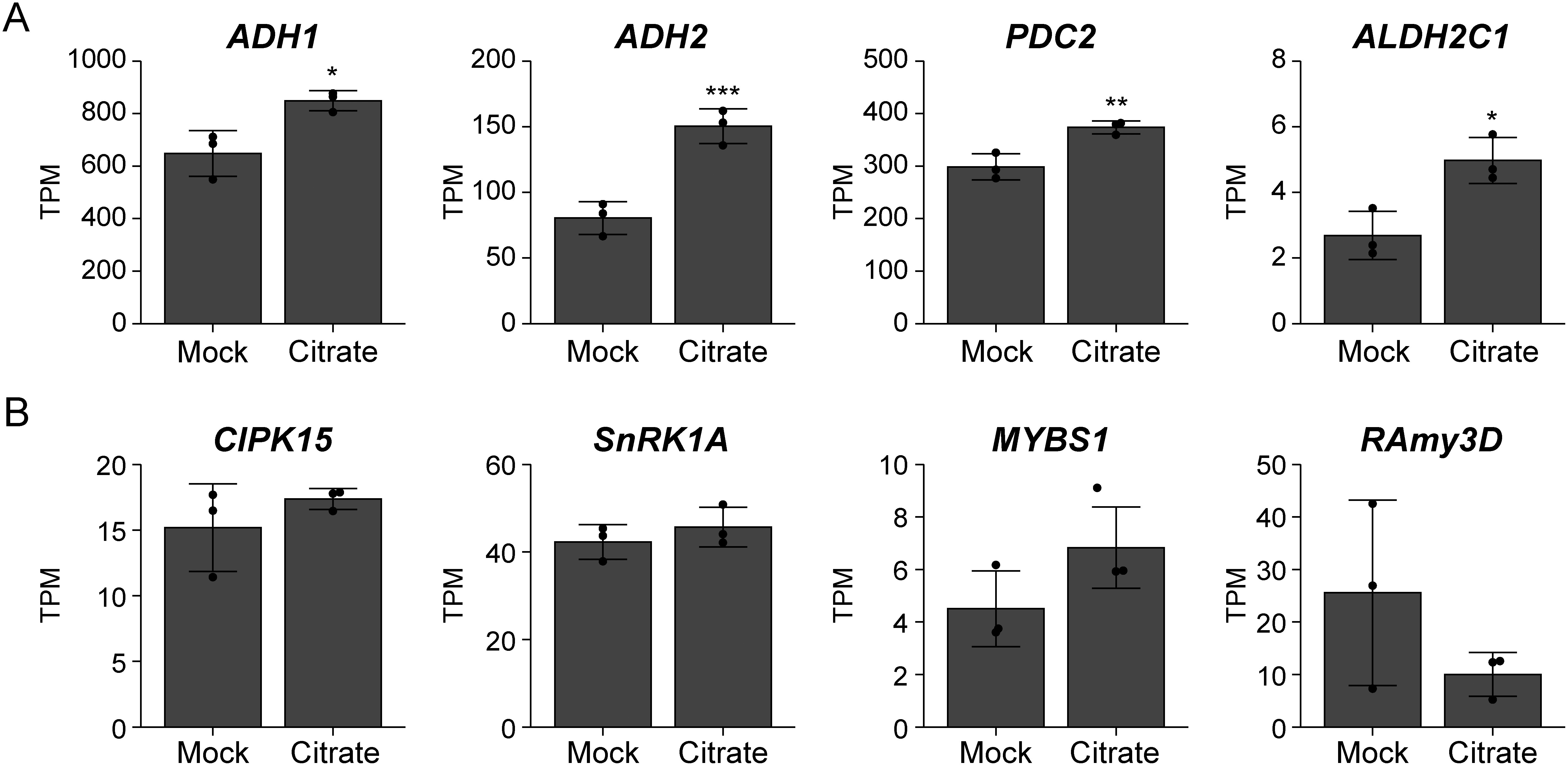
Figure 3. Citrate pretreatment enhances the expression of anaerobic carbohydrate metabolism related genes, but not that of sugar signaling genes, in the embryos of submerged rice seeds. (A, B) Expression levels of anaerobic respiration (A) and sugar signaling (B) marker genes. Daichinohoshi seeds were pretreated with distilled water (Mock) or 10 mM citrate and then incubated in water for 4 days. Gene expression levels were examined in the embryo by RNA-seq and calculated as transcripts per million (TPM). Data represent mean±SD (*n*=3). Asterisks indicate significant differences (* *p*<0.05, ** *p*<0.01, *** *p*<0.001; Student’s *t*-test).

To understand the mechanism responsible for citrate-induced coleoptile elongation in rice, we examined the expression of genes related to anaerobic respiration. Total RNA was isolated from embryos at the early post-germinative growth stage (i.e., 4 days after submergence) and subjected to RT-qPCR analysis. The results indicated that citrate pretreatment enhanced the expression of *ADH1* and *ADH2*, genes encoding key enzymes for anaerobic respiration (Figure 2), suggesting that citrate pretreatment influences transcriptional regulation under submergence. Then, to reveal the global changes in the transcriptome due to citrate pretreatment, we carried out RNA-seq analysis. A total of 752 differentially expressed genes (DEGs) were identified in rice seedlings pretreated with citrate (Supplementary Dataset1). In addition to *ADH1* and *ADH2*, several key enzyme genes related to anaerobic carbohydrate metabolism, such as *pyruvate decarboxylase* *2* (*PDC2*) and *aldehyde dehydrogenase* *2C1* (*ALDH2C1*), were also upregulated in rice embryos pretreated with citrate (Figure 3A and Supplementary Dataset1).

### Starch and soluble sugar contents of submerged rice seeds are not affected by citrate pretreatment

**Figure 4:**
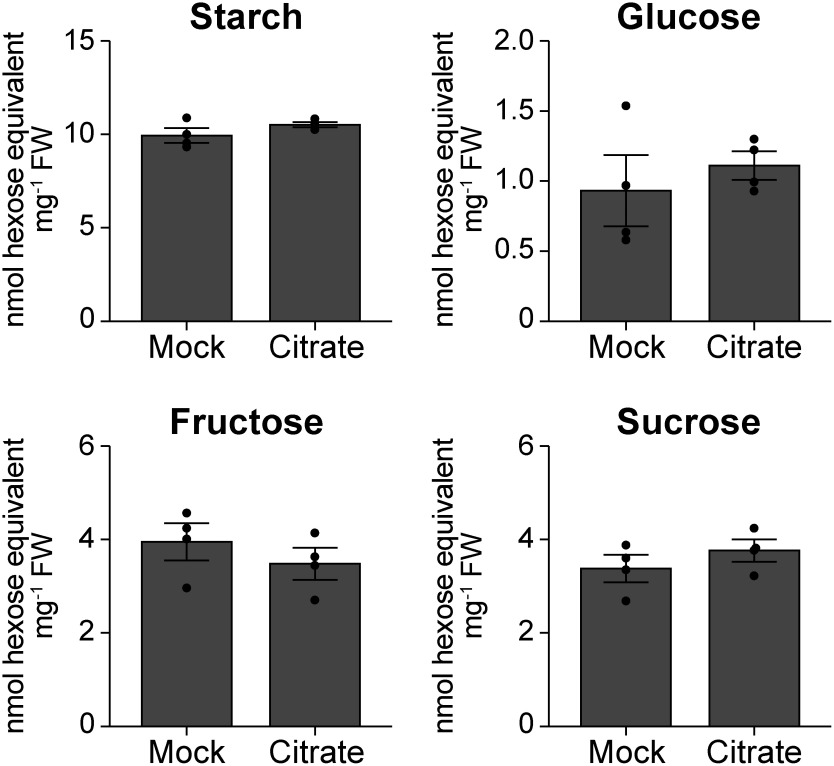
Figure 4. Citrate pretreatment does not affect the starch and soluble sugar contents of rice seeds under submergence. Daichinohoshi seeds were pretreated with distilled water (Mock) or 10 mM citrate and then incubated in water for 2 weeks. Starch and free sugar contents were quantified in the endosperm and embryo, respectively. Data represent mean±SD (*n*=4).

Starch degradation in the endosperm is a critical process that provides soluble sugars for anaerobic respiration under submergence. The SnRK1A-MYBS1-RAmy3D pathway plays a critical role in starch degradation under submergence (Yu et al. 1996). In addition, a previous study showed that the importance of rapid starch degradation in the leaf sheath elongation of rice seedlings germinated under aerobic conditions (Matsukura et al. 1998). However, expression levels of the key starch degradation enzyme gene *RAmy3D* and upstream signaling factor genes *CIPK15*, *SnRK1A*, and *MYBS1* were not significantly influenced by citrate pretreatment (Figure 3B and Supplementary Dataset1). To confirm that starch degradation is not promoted by citrate pretreatment, we measured the cellular contents of starch and soluble sugars in rice seeds. According to gene expression analysis, neither the starch content of the endosperm (Figure 4) nor the soluble sugar (glucose, fructose, or sucrose) content of the embryo was affected by citrate pretreatment (Figure 4).

### Citrate pretreatment alters the expression of phenylpropanoid biosynthesis and ROS regulation related genes

**Figure 5:**
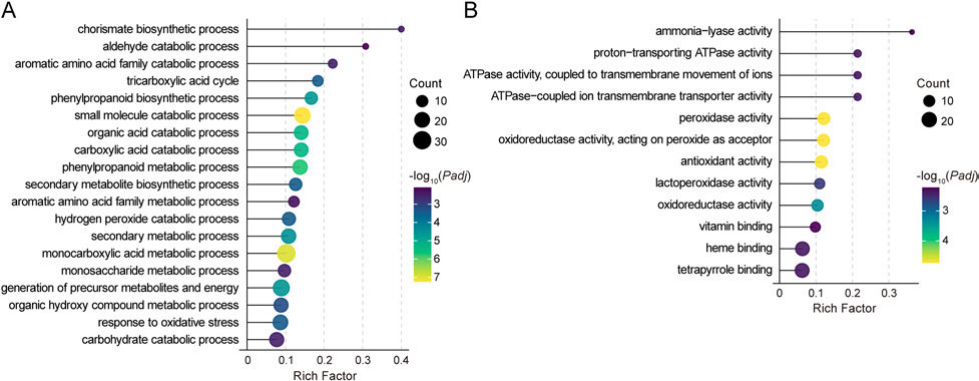
Figure 5. Gene Ontology (GO) enrichment analysis of differentially expressed genes (DEGs) identified in the embryos of submerged rice seeds pretreated with citrate. (A, B) GO terms in the biological process (A) and molecular function (B) categories significantly enriched among the DEGs.

**Figure 6:**
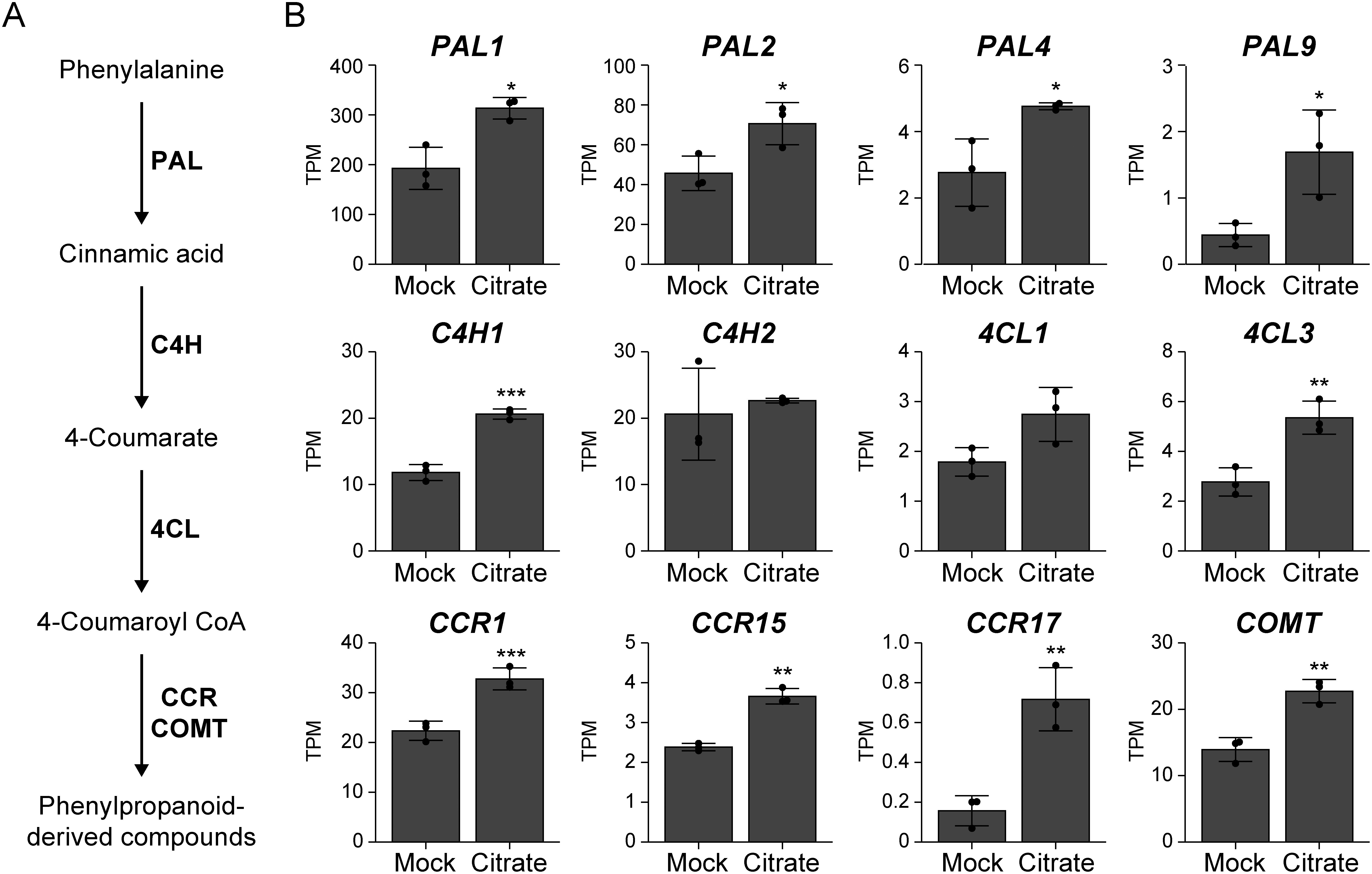
Figure 6. Citrate pretreatment activates the expression of phenylpropanoid biosynthesis genes in the embryos of submerged rice seeds. (A) Schematic representation of the phenylpropanoid biosynthetic pathway. (B) Expression levels of phenylpropanoid biosynthesis genes. Daichinohoshi seeds were pretreated with distilled water (Mock) or 10 mM citrate and then incubated in water for 4 days. Gene expression levels were examined in embryos by RNA-seq and calculated as transcripts per million (TPM). Data represent mean±SD (*n*=3). Asterisks indicate significant differences (* *p*<0.05, ** *p*<0.01, *** *p*<0.001; Student’s *t*-test).

To reveal the potential effect of citrate pretreatment on the growth of submerged rice seedlings, we performed Gene Ontology (GO) analysis of DEGs. The results suggested that not only cellular respiration and carbohydrate metabolism but also GO related to aromatic amino acid (AAA) and phenylpropanoid biosynthesis were enriched as citrate pretreatment responsive genes (Figure 5A). DEGs related to chorismate biosynthetic process, an initial enzymatic step in the shikimate pathway (Sato et al. 2006), included *3-deoxy-D-arabino-heptulosonate-7-phosphate synthetase 1* (*DAHPS1*) (Supplementary Dataset1). Additionally, the expression of genes related to the central phenylpropanoid pathway, such as *phenylalanine ammonia lyases* (*PALs*) (Tonnessen et al. 2015), *cinnamate 4-hydroxylase 1* (*C4H1*) (Koshiba et al. 2013), *4-coumarate:coenzyme A ligase 3* (*4CL3*) (Gui et al. 2011), *cinnamoyl-CoA reductases* (*CCRs*) (Park et al. 2017), and *caffeic acid O-methyltransferase* (*COMT*) (Shimizu et al. 2012), was activated by citrate pretreatment (Figure 6A, B). AAA and AAA-derived secondary metabolites (called plant specialized metabolites) largely contribute to abiotic stress tolerance in plants (Maeda and Dudareva 2012). Phenylpropanoids include phenylalanine- and tyrosine-derived secondary metabolites such as lignin, suberin, anthocyanins, and flavonols. Lignin and suberin are secondary cell wall components that enhance cell wall strength. The synthesis of lignin and suberin is activated under submergence, and their accumulation in the root cell wall creates the ROL barrier, which prevents radial oxygen loss (Colmer et al. 2019). In addition, a recent study found that an intermediate metabolite, cinnamic acid, is involved in the regulation of coleoptile elongation in submerged rice (Vlaminck et al. 2022). Thus, our results and the results of Vlaminck et al. (2022) suggest a possibility that citrate pretreatment enhances coleoptile elongation through the increased cell wall strength and/or reduction of oxygen loss by upregulating the synthesis of several phenylpropanoids including lignin, suberin, and cinnamic acid.

Additionally, GO enrichment analysis based on “molecular function” indicated that the expression of a set of peroxidase (Prx)-encoding genes was influenced by citrate pretreatment (Figure 5B). We found that the expression of class III Prx family genes was especially increased by citrate pretreatment (Supplementary Figure S3). The Prx enzyme plays an essential role in reactive oxygen species (ROS) homeostasis (Francoz et al. 2015; Tsukagoshi et al. 2010). Class III Prx is a plant-specific secretory enzyme located in the apoplast that plays a crucial role in lignification, cell elongation, and seed germination (Kidwai et al. 2019, 2020; Lee 1977; Shigeto and Tsutsumi 2016). Recent transcriptome and *cis*-acting element analyses in sugarcane suggested the involvement of class III Prx family genes in hypoxia stress responses (Shang et al. 2023). Besides, Qiao et al. (2024) showed that the plant hormone ethylene upregulates the expression of class III Prx genes to promote coleoptile elongation in rice (Qiao et al. 2024). These findings suggest that class III Prx enzymes might be involved in the citrate-induced enhancement of coleoptile elongation.

It will be important to elucidate the effect of citrate pretreatment on phenylpropanoid metabolism, cell wall lignification, and ROS levels in rice seedlings under submergence. Exploring additional ELM metabolites capable of promoting coleoptile elongation will provide meaningful insights into the underlying mechanism. Besides, citrate and organic acid as well as ELM treatments could affect cellular and extracellular pH which influences gene expression and plant growth (Tsai and Schmidt 2021). A recent study revealed that plants sense extracellular pH by cell surface peptide-receptor complex, modulating root growth and plant immunity (Liu et al. 2022). Further analysis considering the pH effect on anaerobic respiration and coleoptile elongation might be interesting. Coleoptile elongation is an important physiological process, and optimizing the priming effect of citrate pretreatment on rice seeds could improve the efficiency of rice cultivation via direct seeding and thus contribute to sustainable agriculture.

## Accession numbers

The RNA-seq data have been deposited in the DDBJ database (https://www.ddbj.nig.ac.jp) (Accession number: PRJDB19593). Gene accession numbers used in this study are summarized in Supplementary Dataset1.

## Description of supplementary files

Supplementary Figure S1. Pretreatment of Nipponbare seeds with ELM promotes coleoptile elongation under submergence.

Supplementary Figure S2. Pretreatment of Daichinohoshi seeds with ELM promotes coleoptile elongation under submergence.

Supplementary Figure S3. Citrate pretreatment enhances the expression of class III peroxidase (Prx) genes in the embryos of submerged rice seeds.

Supplementary Table S1. List of primers used in this study.

Supplementary Dataset1. List of DEGs identified by RNA-seq analysis and of accession numbers used in this study.
