# Supplementary material for: Citrate pretreatment promotes rice (Oryza sativa L.) coleoptile elongation under submergence
Source: Plant Biotechnol (Tokyo). 2025 Mar 25;42(1):57–64. doi: 10.5511/plantbiotechnology.24.1220a (PMC12622906; doi:10.5511/plantbiotechnology.24.1220a)
Supplement: Supplementary Data [file plantbiotechnology-42-1-24.1220a-s001.pdf]

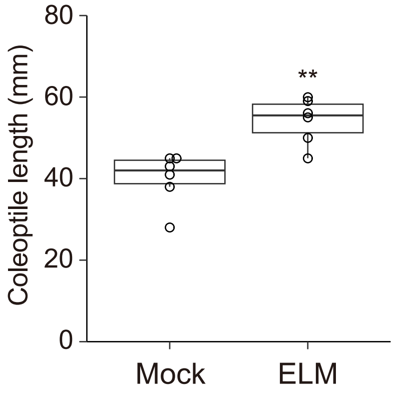


**Supplementary Figure S1.** Pretreatment of Nipponbare seeds with ELM promotes coleoptile elongation under submergence.

Nipponbare seeds were pretreated with distilled water (Mock) or diluted ELM solution for 4 days and then incubated in distilled water for 2 weeks. In each box, the top and bottom lines represent the upper and lower quartiles, respectively; middle horizontal line represents the median (n = 6); and whiskers indicate 1.5 times the interquartile range. Asterisks indicate significant differences (**$p < 0.01$; Student's *t*-test).

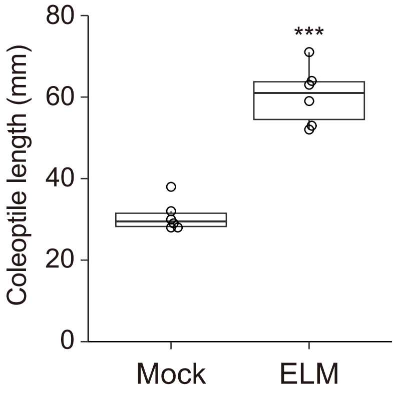


**Supplementary Figure S2.** Pretreatment of Daichinohoshi seeds with ELM promotes coleoptile elongation under submergence.

Daichinohoshi seeds were pretreated with distilled water (Mock) or diluted ELM solution for 4 days and then incubated in distilled water for 2 weeks. In each box, the top and bottom lines represent the upper and lower quartiles, respectively; middle horizontal line represents the median (n = 6); and whiskers indicate 1.5 times the interquartile range. Asterisks indicate significant differences (***$p < 0.001$; Student's *t*-test).

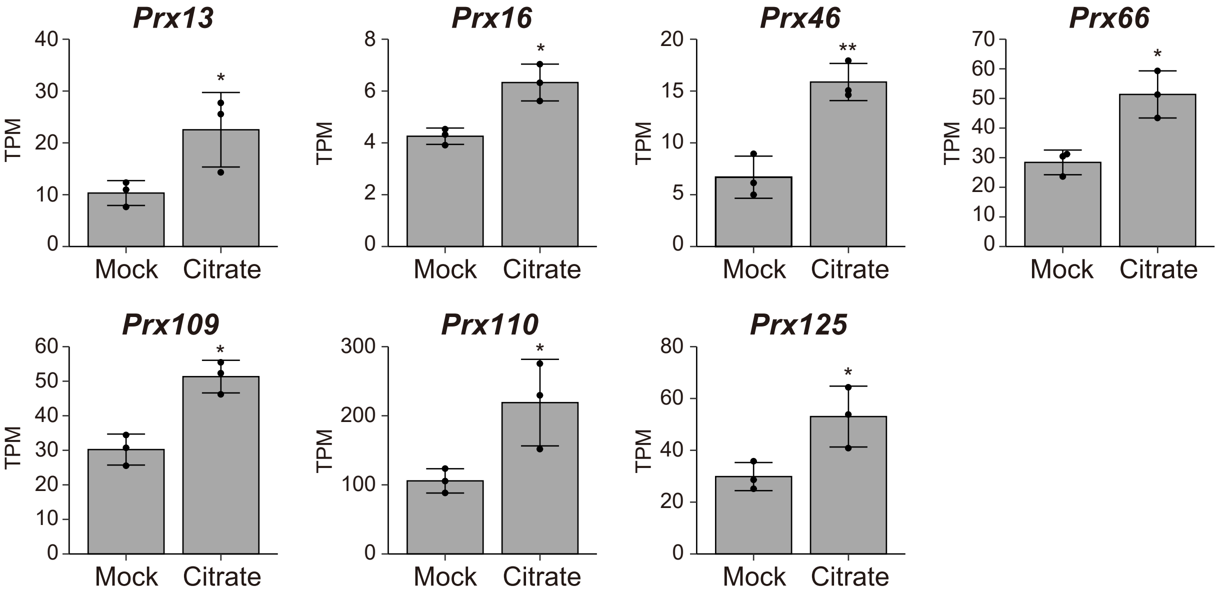


**Supplementary Figure S3.** Citrate pretreatment enhances the expression of class III peroxidase (Prx) genes in the embryos of submerged rice seeds.

Daichinohoshi seeds were pretreated with distilled water (Mock) or 10 mM citrate and then incubated in water for 4 days. Gene expression levels were analyzed in embryos by RNA-seq and calculated as transcripts per million (TPM). Data represent mean ± SD (n = 3). Asterisks indicate significant differences (*$p < 0.05$, **$p < 0.01$; Student' s *t*-test).

Supplementary Table S1. Primers used in this study

| Primer name | Sequence (5'-3') |
|---|---|
| OsUBC5b_qPCR 5-1 | CTCAAGGACCTGCAGAAGGA |
| OsUBC5b_qPCR 3-1 | ATGGACCCATCAGTGTTGC |
| OsADH1_qPCR 5-1 | ACGAGTTTCAGTTCGTCACCCTCT |
| OsADH1_qPCR 3-1 | AACCACAACTCGAGCGCACAAATC |
| OsADH2_qPCR 5-1 | CTCAACGAGAAGACGCTCAA |
| OsADH2_qPCR 3-1 | TGCGTGATGAACTTCTCCAG |
